# Incidence of intra-procedural complications according to the timing of endovascular treatment in ruptured intracranial aneurysms

**DOI:** 10.3389/fneur.2022.1096651

**Published:** 2023-01-11

**Authors:** Chiara Gaudino, Stefania Elena Navone, Valerio Da Ros, Laura Guarnaccia, Giovanni Marfia, Patrizia Pantano, Simone Peschillo, Fabio Maria Triulzi, Francesco Biraschi

**Affiliations:** ^1^Department of Neuroradiology, Azienda Ospedaliero-Universitaria Policlinico Umberto I, Rome, Italy; ^2^Department of Neuroradiology, Fondazione IRCCS Ca'Granda Ospedale Maggiore Policlinico, Milan, Italy; ^3^Laboratory of Experimental Neurosurgery and Cell Therapy, Neurosurgery Unit, Fondazione IRCCS Ca' Granda Ospedale Maggiore Policlinico, Milan, Italy; ^4^Diagnostic Imaging Unit, Department of Biomedicine and Prevention, University of Rome “Tor Vergata”, Rome, Italy; ^5^Clinical Pathology Unit, Aerospace Medicine Institute “A. Mosso”, Italian Air Force, Milan, Italy; ^6^Department of Human Neurosciences, Sapienza University of Rome, Rome, Italy; ^7^IRCCS Neuromed, Pozzilli, IS, Italy; ^8^Casa di Cura Quisisana, Rome, Italy; ^9^Department of Pathophysiology and Transplantation, Università degli Studi di Milano, Milan, Italy

**Keywords:** ruptured intracranial aneurysm, endovascular treatment, intra-procedural complications, timing of endovascular treatment, subarachnoid hemorrhage

## Abstract

**Background:**

Although endovascular treatment of ruptured intracranial aneurysms is well-established, some critical issues have not yet been clarified, such as the effects of timing on safety and effectiveness of the procedure. The aim of our study was to analyze the incidence of intra-procedural complications according to the timing of treatment, as they can affect morbidity and mortality.

**Materials and methods:**

We retrospectively analyzed all patients who underwent endovascular treatment for ruptured intracranial aneurysms at three high flow center. For all patients, imaging and clinical data, aneurysm's type, mean dimension and different treatment techniques were analyzed. Intra-procedural complications were defined as thrombus formation at the aneurysm's neck, thromboembolic events, and rupture of the aneurysm. Patients were divided into three groups according to time between subarachnoid hemorrhage and treatment (<12 h hyper-early, 12–36 h early, and >36 h delayed).

**Results:**

The final study population included 215 patients. In total, 84 patients (39%) underwent hyper-early, 104 (48%) early, and 27 (13%) delayed endovascular treatment. Overall, 69% of the patients were treated with simple coiling, 23% with balloon-assisted coiling, 1% with stent-assisted coiling, 3% with a flow-diverter stent, 3% with an intrasaccular flow disruptor device, and 0.5% with parent vessel occlusion. Delayed endovascular treatment was associated with an increased risk of total intra-procedural complications compared to both hyper-early (*p* = 0.009) and early (*p* = 0.004) treatments with a rate of complications of 56% (vs. 29% in hyper-early and 26% in early treated group—*p* = 0.011 and *p* = 0.008). The delayed treatment group showed a higher rate of thrombus formation and thromboembolic events. The increased risk of total intra-procedural complications in delayed treatment was confirmed, also considering only the patients treated with simple coiling and balloon-assisted coiling (*p* = 0.005 and *p* = 0.003, respectively, compared to hyper-early and early group) with a rate of complications of 62% (vs. 28% in hyper-early and 26% in early treatments—*p* = 0.007 and *p* = 0.003). Also in this subpopulation, delayed treated patients showed a higher incidence of thrombus formation and thromboembolic events.

**Conclusions:**

Endovascular treatment of ruptured intracranial aneurysms more than 36 h after SAH seems to be associated with a higher risk of intra-procedural complications, especially thrombotic and thromboembolic events.

## Introduction

Subarachnoid hemorrhage (SAH) due to aneurysmal rupture is associated with important mortality and morbidity (1, 2). Ruptured intracranial aneurysms may be treated surgically or endovascularly. The multicenter randomized controlled International Subarachnoid Aneurysm Trial (ISTAT) compared the surgical clipping and endovascular simple coiling of ruptured intracranial aneurysms (1, 2). The ISTAT study provided important information regarding the indications of the different types of treatment and their clinical outcomes, concluding that patients treated endovascularly had a better clinical outcome at 1 year and a similar clinical outcome at 5 years (2). Although the endovascular treatment for ruptured intracranial aneurysms is well-established and widely used, the effect of the treatment's timing on its safety is poorly understood. In 2012, the American Heart Association (AHA) together with the American Stroke Association (ASA) recommended the treatment of ruptured aneurysms as early as feasible to reduce the risk of rebleeding (3). In 2013, the European Stroke Organization underlined that the treatment should be aimed at least within 72 h after onset of first symptoms (4). Only few studies have focused on the best timing of treatment after SAH and most of them included only surgical clipping or both surgical and endovascular treatments (5–16). The definition of hyper-early, early, and delayed treatment varies widely in the different studies and their results are contradictory. By comparing patients treated within 48 h and patients treated between 48 h and 30 days after symptom onset, Baltsavias et al. (12) showed that the timing of endovascular treatment did not influence the periprocedural morbidity and the clinical outcome at 6 months. Philips et al. (13) demonstrated that in their mixed surgical and endovascular series, the patients treated within 24 h after symptom onset had a better clinical outcome at 6 months. These data were confirmed by Consoli et al. (14), who demonstrated that hyper-early endovascular treatment within 12 h after SAH is not associated with a lower morbidity or a better clinical outcome with respect to treatment after 12 h. Recently, Buscot et al. (15) and Wu et al. (16) showed that best clinical outcomes were achieved treating the patients at ~12.5 h.

Despite these studies, the effects of timing of endovascular procedure, not only on the medium-term clinical outcome, but also on intra-procedural complications, which may affect morbidity and mortality, in patients with ruptured intracranial aneurysms, are still unclear. Thus, the aim of this multicenter study was to analyze the incidence of intra-procedural complications according to the timing of endovascular treatment in a large series of patients who underwent endovascular treatment for ruptured intracranial aneurysms.

## Materials and methods

### Study design and patient population

We retrospectively analyzed 226 patients treated endovascularly for ruptured intracranial aneurysms at three high flow centers in 3 consecutive years. Patients were not randomized.

### Clinical and imaging data

At admission, all patients underwent clinical evaluation, brain computed tomography (CT), and a CT angiography. Then, each patient underwent a digital subtraction angiography (DSA) on a flat panel unit with 3D rotational acquisition. Each case was evaluated by the neurosurgeon and the interventional neuroradiologist to decide the choice of treatment: endovascular treatment was preferred except in big hematomas with mass effect and in case of arterial branches originating from the aneurysm's sac. The timing of endovascular treatment was left up to the discretion of treatment teams. Imaging and clinical data of patients treated endovascularly were retrospectively analyzed by three experienced interventional neuroradiologists (with more than 8 years of clinical practice; C.G., F.B., and V.D.R.) using the modified Fisher's and Hunt and Hess scale. For each patient, aneurysm's location and type (saccular with narrow or wide neck, dissecting, and blister), mean aneurysm's dimension, and different endovascular treatment techniques (simple coiling, balloon-assisted coiling, stent-assisted coiling, placement of flow-diverter stents, or intrasaccular flow-disruptor and parent vessel occlusion) were considered. Intra-procedural complications were defined as thrombus formation at the aneurysm's neck, thromboembolic events, and rupture of the aneurysm. For each patient, general risk factors such as hypertension, diabetes mellitus, dyslipidemia, anticoagulation, and antiplatelet therapy before SAH were considered. Patients were divided into three groups according to time between SAH and treatment: <12 h = hyper-early, 12–36 h = early, and >36 h = delayed. The interventional neuroradiologists were blinded regarding the timing of endovascular treatment, while analyzing the clinical and imaging data.

### Sample size calculation

The primary objective of our study was to investigate the incidence of intra-procedural complications according to the timing of treatment, as they can affect morbidity and mortality in patients with SAH. Considering three groups (<12 h = hyper-early, 12–36 h = early, and >36 h = delayed), an alpha error of 0.05, a power of 0.9, and an effect size of 0.25, we calculated that a minimum sample size of 207 patients with SAH is required (Gpower, Heinrich-Heine-Universität Düsseldorf).

### Statistical analysis

Demographic, clinical, imaging, and angiographic data were analyzed using SPSS software (IBM SPSS Statistics for Windows, version 26.0, IBM Corporation, Armonk, NY, USA, RRID:SCR_002865). Continuous variables were presented as median and interquartile range (IQR), depending on the distribution of data. Categorical variables were presented as counts and percentages. Demographic and clinical parameters were tested for normality using the Kolmogorov–Smirnov and Shapiro–Wilk tests. When normally distributed, a two-way ANOVA was used to compare, simultaneously, the different variables between the three treatment groups; otherwise, when not-normally distributed, the variables were compared by the Kruskal–Wallis test for the independent samples. Chi-square test was used to perform the statistical analysis in global population and within the three different treatment groups. The odd ratio (OR) was calculated using the logistic regression analysis.

Also, multivariate analysis with logistic regression was performed within the different treatment groups. To select the variables for the multivariate analysis, the total study population was divided into two groups according to the presence or absence of intra-procedural complications and univariate analysis was conducted to identify the association between baseline characteristics and intra-procedural complications. Qualitative variables were analyzed by chi-square test. Quantitative variables, when normally distributed, were analyzed by the two-tailed Student's *t*-test; otherwise, when not normally distributed, no-parametric Mann–Whitney test was performed.

The tests were considered statistically significant when *p* < 0.05.

## Results

We retrospectively analyzed 226 consecutive patients treated endovascularly for ruptured intracranial aneurysms; 11 patients were excluded because they were treated more than 4 days after SAH. The final study population included 215 patients with 216 aneurysms (74 men and 141 women; mean age: 58.2 ± 12.5 years, range: 29–91 years). Clinical and imaging data at admission are summarized in Table 1. All patients were treated as soon as possible after admission: 84 patients (39%) were treated in the first 12 h, 104 patients (48%) between 12 and 36 h, and 27 patients (13%) more than 36 h after aneurysm's rupture.

**Table 1:** Demographic and clinical parameters in hyper-early, early, and delayed treatment groups.

		** <12 h**	**12–36 h**	**>36 h**	**Tot**	***p*-value**
Patients, *n*° (%)		84 (39)	104 (48)	27 (13)	215 (100)	n.s
Age, median (IQR), year		55 (48–64)	60 (50–69)	56 (45–70)	58 (49–57)	n.s.
Sex M/F, *n*° (%)		28/56 (33/67)	31/73 (70/30)	15/12 (44/56)	113/102 (52/48)	n.s.
Treatment time from SAH, mean ± std dev, h		7.2 ± 2.4	19.01 ± 5.7	59.8 ± 17.25	19.6 ± 17.9	n.s.
Hunt Hess, *n*° (%)	1	13 (15.5)	31 (29.8)	10 (37.0)	54 (25.1)	*p* <0.05
	2	32 (38.1)	38 (36.5)	11 (40.8)	81 (37.7)	n.s.
	3	17 (20.2)	18 (17.3)	3 (11.1)	38 (17.7)	n.s.
	4	17 (20.2)	14 (13.5)	3 (11.1)	34 (15.8)	n.s.
	5	5 (6)	3 (2.9)	0 (0)	8 (3.7)	n.s.
Modified Fisher, *n*° (%)	0	0 (0)	0 (0)	1 (3.7)	1 (0.5)	n.s.
	1	11 (13.1)	25 (24.0)	11 (40.7)	47 (21.9)	n.s.
	2	29 (34.5)	37 (35.6)	5 (18.5)	71 (33.0)	n.s.
	3	18 (21.4)	16 (15.4)	5 (18.5)	39 (18.1)	n.s.
	4	26 (30.9)	26 (25.0)	5 (18.5)	57 (26.5)	n.s.
Rebleeding before treatment, *n*° (%)		6 (7.1)	4 (3.8)	2 (7.4)	12 (5.6)	n.s.
Location, *n*° (%)	ICA	14 (16.6)	24 (22.8)	6 (22.2)	44 (20.3)	n.s.
	PcomA	12 (14.3)	11 (10.5)	4 (14.8)	27 (12.5)	n.s.
	MCA	12 (14.3)	10 (9.5)	4 (14.8)	26 (12.0)	n.s.
	ACA	16 (19.0)	10 (9.5)	0 (0)	26 (12.0)	n.s.
	AcomA	24 (28.6)	36 (34.3)	10 (37.1)	70 (32.4)	n.s.
	VA	0 (0)	4 (3.8)	0 (0)	4 (1.9)	n.s.
	PICA	1 (1.2)	7 (6.7)	3 (11.1)	11 (5.1)	n.s.
	BA	4 (4.8)	2 (1.9)	0 (0)	6 (2.8)	n.s.
	SCA	0 (0)	1 (1)	0 (0)	1 (0.5)	n.s.
	PCA	1 (1.2)	0 (0)	0 (0)	1 (0.5)	n.s.
Dimension, median (IQR) (mm)		5.25 (3.97–7.15)	4.7 (3.7–6.7)	4.0 (3.3–5.3)	4.8 (3.7–6.5)	n.s.
Type, *n*° (%)	Saccular with narrow neck	42 (50)	58 (55.2)	11 (40.8)	111 (51.4)	n.s.
	Saccular with wide neck	37 (44.0)	32 (30.5)	9 (33.3)	78 (36.1)	n.s.
	Blister	0 (0)	3 (2.9)	4 (14.8)	7 (3.2)	*p* ≤ 0.01
	Dissecting	5 (6)	12 (11.4)	3 (11.1)	20 (9.3)	n.s.
All complication, *n*° (%)		24 (28.6)	27 (26.0)	15 (55.6)	66 (30.7)	*p* ≤ 0.01
Rupture, *n*° (%)		8 (9.5)	5 (4.8)	4 (14.8)	17 (7.9)	n.s.
Thromboembolic events, *n*° (%)		18 (21.4)	26 (25.0)	11 (40.7)	55 (25.6)	*p* <0.05
Event resolution, *n*° (%)		10 (41.7)	15 (55.6)	8 (53.3)	33 (50)	n.s.
Secondary lesion, *n*° (%)		8 (9.5)	12 (11.5)	4 (14.8)	24 (11.2)	n.s.
Endovascular treatment, *n*° (%)	Simple coiling	56 (66.6)	78 (74.3)	15 (55.6)	149 (69.0)	n.s.
	Balloon-assisted coiling	26 (31.0)	18 (17.1)	6 (22.2)	50 (23.1)	n.s.
	Stent and coiling	1 (1.2)	0 (0)	2 (7.4)	3 (1.4)	n.s.
	Flow-diverter stent	0 (0)	5 (4.8)	1 (3.7)	6 (2.8)	n.s.
	WEB	1 (1.2)	3 (2.8)	3 (11.1)	7 (3.2)	*p* <0.05
	Parent vessel occlusion	0 (0)	1 (1.0)	0 (0)	1 (0.5)	n.s.
Anticoagulation therapy during treatment, *n*° (%)		55 (65.5)	70 (67.3)	19 (70.3)	144 (67.0)	n.s.
	i.v. bolus	26 (31.0)	45 (43.3)	11 (40.7)	82 (38.1)	n.s.
	Infusion through catheter	8 (9.5)	13 (12.5)	5 (18.5)	26 (12.1)	n.s.
	Infusion through microcatheter	21 (25.0)	12 (11.5)	3 (11.1)	36 (16.7)	n.s.
Antiplatelet therapy during treatment, *n*° (%)		12 (14.3)	13 (12.5)	7 (25.9)	32 (14.9)	n.s.
Previous anticoagulation or antiplatelet, *n*° (%)		9 (10.7)	20 (19.2)	5 (18.5)	34 (15.8)	n.s.
Risk factors, *n*° (%)	Hypertension	41 (48.8)	54 (51.9)	17 (63.0)	112 (52.1)	n.s.
	Diabetes mellitus	5 (6.0)	14 (13.5)	4 (14.8)	23 (10.7)	n.s
	Dyslipidemia	7 (8.3)	22 (21.2)	7 (25.9)	36 (16.7)	n.s

*p*-value comparing hyper-early, early, and delayed treatment groups.

n.s., not significant; ICA, internal carotid artery; PcomA, posterior communicating artery; MCA, middle cerebral artery; ACA, anterior cerebral artery; AcomA, anterior communicating artery; VA, vertebral artery; PICA, posterior inferior cerebellar artery; BA, basilar artery; SCA, superior cerebellar artery; PCA, posterior cerebral artery.

Computed tomography scan at admission was positive for SAH in 214 patients. In one patient, SAH was diagnosed with lumbar puncture. In 192 patients (89%), the aneurysm was located in the anterior circulation and in 23 (11%), the aneurysm was located in the posterior circulation. The ruptured aneurysm was saccular with narrow neck in 111 cases (52%), saccular with wide neck in 78 cases (36%), dissecting in 20 cases (9%), and blister in seven cases (3%).

**Figure 1:**
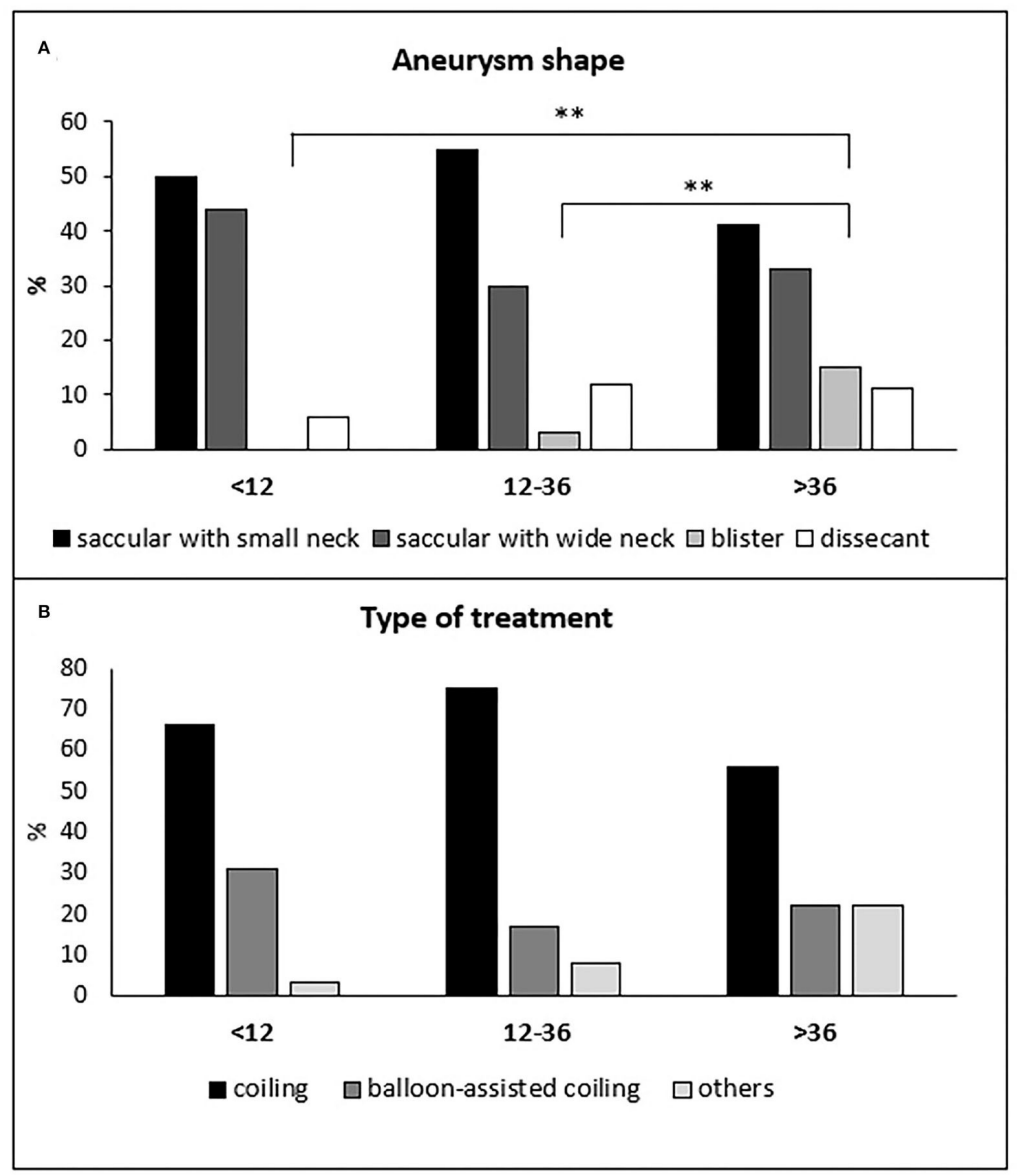
Distribution of the different aneurysm shapes **(A)** and types of treatments **(B)** in the hyper-early, early, and delayed treatment groups of patients. **(A)** **p ≤ 0.01.

The three treatment groups did not differ significantly for age distribution, risk factors, and Fisher's grade (Table 1). Hunt and Hess grade 1 was more frequent in the delayed than in the hyper-early treated group (10 of 27 patients −37% vs, 13 of 84 patients −16%; *p* = 0.049). Blisters aneurysms were more frequent in the delayed than in hyper-early and early groups (4 of 27 patients −15% vs. 0 of 84 patients −0% and 3 of 104 patients 3%, respectively; *p* = 0.01; Figure 1A). Pre-treatment rebleeding was observed in 12 patients (5.6%): six of 84 patients (7.1%), four of 104 patients (3.8%), and two of 27 patients (7.4%) in the hyper-early, early, and delayed groups, respectively (*p* = n.s.).

In total, 149 of 215 patients (69%) were treated with simple coiling, 50 patients (23%) with balloon-assisted coiling, three patients (1%) with stent-assisted coiling, six patients (3%) with the deployment of a flow-diverter stent, seven patients (3%) with an intrasaccular flow disruptor device (Woven EndoBridge—WEB) and one patients (0.5%) with parent vessel occlusion. The WEB device was more frequently used in the delayed group compared to the hyper-early group (3 of 27 patients −11% vs. 1 of 84 patients −1.2%; *p* = 0.048; Figure 1B). The three treatment groups did not differ significantly either for the others treatment techniques or for the anticoagulation or antiplatelet therapy during or previous endovascular treatment (Table 1). Activated clotting time (ACT) was not tested during the intervention.

**Figure 2:**
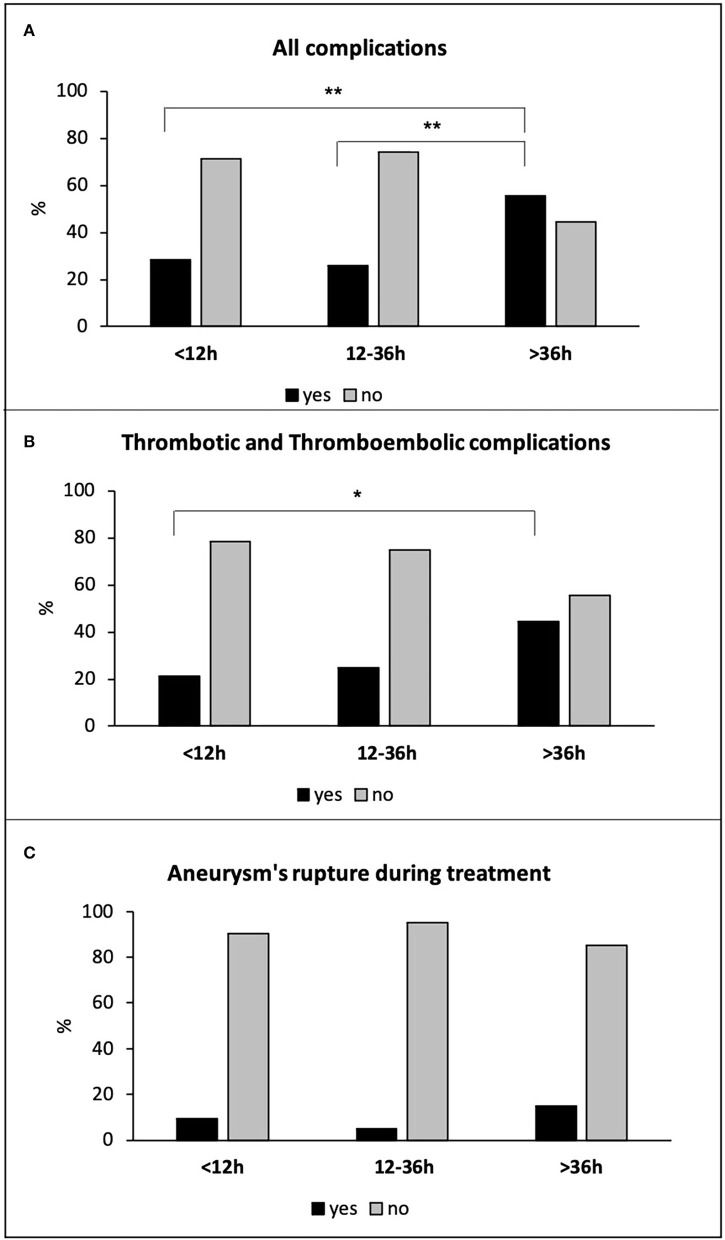
All complications **(A)**, thrombotic and thromboembolic complications **(B)**, and aneurysm's rupture during treatment **(C)** in all hyper-early, early, and delayed treated patients. *p < 0.05; **p ≤ 0.01.

Patients treated after 36 h showed a significantly higher rate of total intra-procedural complications (15 of 27 patients −56%) compared to both hyper-early (24 of 84 patients −29%) and early (27 of 104 patients −26%) groups (*p* = 0.011 and *p* = 0.008, respectively; Figure 2A). Accordingly, delayed treatment was associated with an increased risk of total complications with respect to both hyper-early and early treatment groups (OR: 3.315, 95% CI: 1.351–8.137, *p* = 0.009, and OR: 3.565; 95% CI: 1.484–8.565, *p* = 0.004, respectively). More in detail, the delayed treatment group showed a higher rate of thrombus formation and thromboembolic events (12 of 27 patients −44%), compared to hyper-early (17 of 84 patients −21%; *p* = 0.037) and early (26 of 104 patients −25%; *p* = n.s.) treated patients (Figure 2B). The three treatment groups did not differ significantly for the incidence of aneurysm's rupture during endovascular treatment: seven of 84 patients −10%; five of 104 patients −5%; four of 27 patients −15%, in the hyper-early, early, and delayed treatment groups, respectively (*p* = n.s.; Figure 2C). The complication was resolved in 33 of 66 cases (50%) and only 24 of 215 patients (11%) presented a secondary lesion related to the event.

Univariate logistic regression analysis comparing patients with intra-procedural complications and patients with no complications revealed that treatment time from SAH (χ^2^; *p* = 0.041), Hunt and Hess grade (χ^2^; *p* = 0.036), and heparin during procedure (χ^2^; *p* = 0.037) were significantly associated with intra-procedural complications. Variables with moderate to high association, without reaching the significance level, were modified Fisher (χ^2^; *p* = 0.315), rebleeding before treatment (χ^2^; *p* = 0.267), antiplatelets and/or anticoagulant therapy at home (χ^2^; *p* = 0.063), and aneurysm's location (χ^2^; *p* = 0.207). Interestingly, multivariate logistic regression analysis, conducted with above-mentioned significant predictors and variables with moderate to high association, revealed that, within the three different treatment groups, only treatment time from SAH was an independent predictor factor for aneurysm treatment complications [OR: 1.757, (95% CI: 1.073–2.879); *p* = 0.025; Table 2].

**Table 2:** Multivariate logistic regression models for identification of clinically relevant predictors of aneurism treatment complications.

**Parameters**	**Adjusted OR**	**95% CI**	***p*-value**
Treatment time from SAH (<12 h, 12–36 h, >36 h)	1.757	1.073–2.879	0.025
Hunt and Hess	1.374	0.976–1.935	0.069
Heparin during procedure	1.737	0.853–3.537	0.128
Modified Fisher	1.059	0.754–1.487	0.742
Rebleeding before treatment	0.350	0.068–1.788	0.207
Aneurysm's location	1.840	0.801–4.227	0.151
Antiplatelets and/or anticoagulant therapy at home	1.915	0.865–4.238	0.109

adjusted OR, adjusted odd ratio; CI, confidence interval; SAH, subarachnoid hemorrhage.

**Figure 3:**
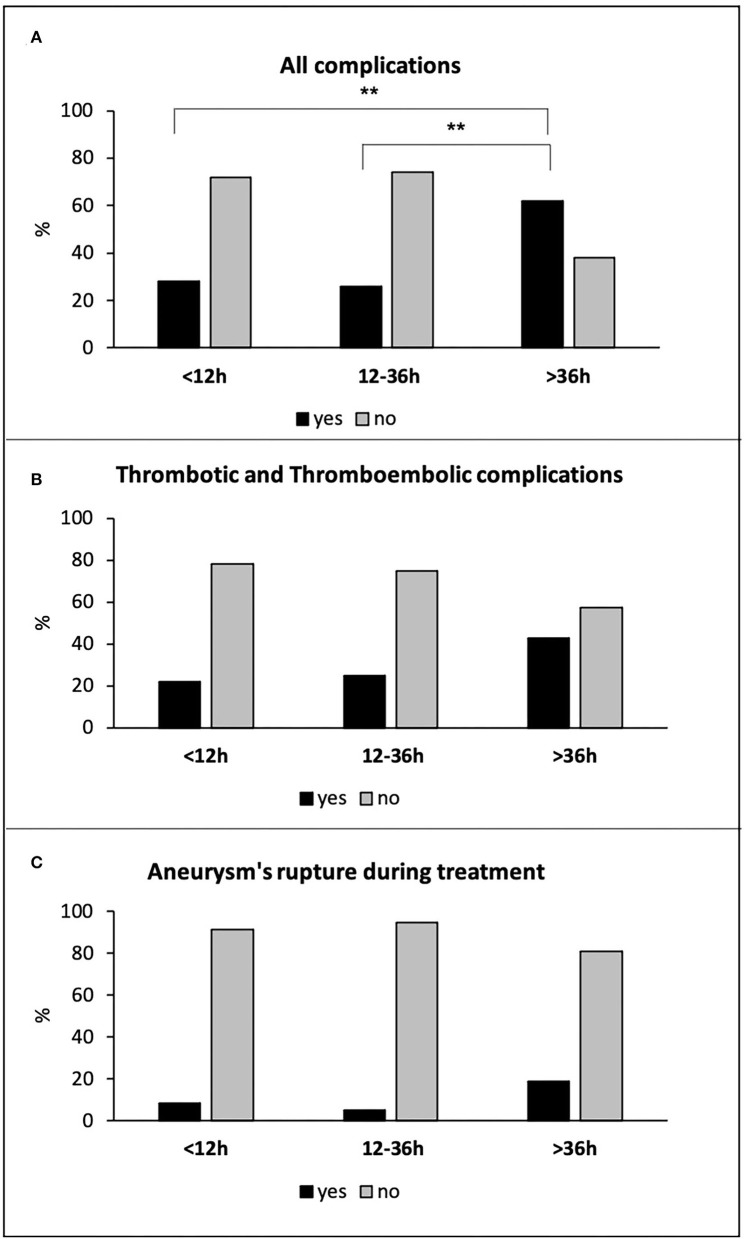
All complications **(A)**, thrombotic and thromboembolic complications **(B)**, and aneurysm's rupture during treatment **(C)** in hyper-early, early, and delayed treated patients with simple coiling and balloon-assisted coiling. **p ≤ 0.01.

Due to the higher risk of thrombus formation and thromboembolic events associated with stent-assisted coiling, flow-diverter stent, and WEB in the acute phase, we analyzed the subpopulation of 199 patients treated with simple coiling (149 patients) and balloon-assisted coiling (50 patients). There were 82 patients (41%) with hyper-early, 96 patients (48%) with early, and 21 patients (11%) with delayed treatments. Considering only the patients treated with simple coiling and balloon-assisted coiling, we have excluded all patients with blister aneurysms, with no difference in the distribution of the other aneurysm types in the three treatment groups. Also, in this subpopulation of patients, the delayed treatment was associated with a higher rate of total intra-procedural complications (13 of 21 patients −62%) compared to both hyper-early (23 of 82 patients −28%; *p* = 0.007), and early (25 of 96 patients −26%; *p* = 0.003) groups (Figure 3A). Delayed treatment was associated with an increased risk of total complications with respect to both hyper-early and early treated groups (OR: 4.168, 95% CI: 1.528–11.375, *p* = 0.005 and OR: 4.615; 95% CI: 1.712–12.441, *p* = 0.003, respectively). Although the delayed treated patients showed a higher frequency in thrombus formation and thromboembolic events (9 of 21 patients −43%) as compared to hyper-early (18 of 82 patients −22%) and early (24 of 96 patients −25%) patients' groups, these differences did not reach statistical significance (Figure 3B). Finally, the three treatment groups did not differ for the incidence of aneurysm's rupture during endovascular treatment: seven of 82 patients −9%; five of 96 patients −5%; and four of 21 patients −19% in the hyper-early, early, and delayed treatment groups, respectively (*p* = n.s.; Figure 3C).

## Discussion

Although endovascular treatment is currently the first-choice treatment in ruptured intracranial aneurysms in most cases (1, 2, 17), some critical issues have not yet been clarified, such as the effects of the timing on safety and effectiveness of the procedure. Our aim was to analyze the incidence of intra-procedural complications in relation to the timing of endovascular treatment, as they can affect morbidity and mortality of these patients.

This study showed that the endovascular treatment of aneurysms more than 36 h after rupture was associated with a significant increased risk of total intra-procedural complications compared to both hyper-early (<12 h) and early (12–36 h) treatments. The delayed treatment group showed particularly a higher rate of thrombus formation and thromboembolic events. The increased risk of total intra-procedural complications in delayed treatment was confirmed, also considering only the subpopulation of patients treated with simple coiling and balloon-assisted coiling. Also in this subpopulation, delayed treated patients showed a higher incidence of thrombus formation and thromboembolic events, compared to hyper-early and early patient's groups. On the other hand, in the total patient's population and in the subpopulation treated with simple coiling or balloon-assisted coiling, no differences were found in the incidence of intra-procedural complications in patients in the hyper-early and early treatment groups.

These data can be explained by the complex platelet aggregation phenomenon observed in patients with SAH (18) and studied in many animals' models (19–27). In most of these studies, the thrombus formation has been evaluated only in the first hours after SAH or at a single time-point. Pisapia et al. (21) examined the microvascular thrombi formation over time in an endovascular perforation mouse model. Using an antithrombin immunostaining, they demonstrated the presence of thrombus in small vessels at 24, 48, 72, and 96 h after SAH with a peak of severity at 48 h. Similarly, Muroi et al. (22) showed a microvascular thrombus formation peak on days 2 and 3 after experimental SAH in the same mouse model using fibrinogen immunostaining. Stein et al. (28) showed similar results in their autopsy series of patients died after SAH, demonstrating that the microclot burden was higher in patients died within 2 days after aneurysm rupture and decreased at days 3 and 4. Many mediators of the inflammation, such as IL-1 and IL-6, and prothrombotic factors, such as platelet-activating factor (PAF), von Willebrand factor (vWF), and β-thromboglobulin, have been involved in the microthrombus formation following SAH (29–32). Hirashima et al. studied the level of PAF in the peripheral blood (30) and in the jugular vein (31) of patients with SAH showing a peak at days 5–9, explaining also the crucial role of platelet aggregation in thrombus formation during endovascular treatment of ruptured aneurysms as demonstrated by Larco et al. (33) in their histopathological study.

Our data are in contrast to the results of Consoli et al. (14), who did not find a difference in the incidence of intra-procedural complications in hyper-early (<12 h), early (12–24 h), and delayed (>24 h) treated patients. This discrepancy may be due to the differences in patients' grouping based on the treatment timing and in complications taken into the consideration between their and our study. While they may have considered only the complications not resolved during the endovascular procedure, we considered all the complications which occurred. In total, 50% of the complications in our series were then resolved endovascularly with only 11% of all patients treated who suffered of a secondary lesion related to the intra-procedural event.

Our study's primary limitation is its retrospective design, which results in a smaller number of patients in the group receiving delayed treatment. On the other side, it might better reflect what actually occurs in daily life, such as more patients in the delayed treatment group having lower Hunt and Hess scores as a result of underestimating the symptoms. The absence of an ACT examination during the endovascular treatment is another limitation.

Neuroinflammation seems to have a crucial role in aneurysm formation and rupture and seems to be involved in vasospasm, hydrocephalus, and headache in patients with SAH (34, 35). It would be interesting to analyze, in a large prospective study, whether a higher level of neuroinflammation is associated with a higher risk of intra-procedural complications during endovascular treatment.

## Conclusions

Endovascular treatment of ruptured intracranial aneurysms more than 36 h after SAH seems to be associated with a higher risk of intra-procedural complications, especially thrombotic and thromboembolic events. If available, treatment should be achieved before 36 h after bleeding. A hyper-early treatment performed in the first 12 h after SAH seems to be as safe as between 12 and 36 h.

## Data availability statement

The raw data supporting the conclusions of this article will be made available by the authors, without undue reservation.

## Ethics statement

Ethical review and approval was not required for the study on human participants in accordance with the local legislation and institutional requirements. Written informed consent from the patients/participants or patients/participants' legal guardian/next of kin was not required to participate in this study in accordance with the national legislation and the institutional requirements.

## Author contributions

All authors listed have made a substantial, direct, and intellectual contribution to the work and approved it for publication.
